# Texture Evaluation and In Vivo Oral Tactile Perceptions of Cooked Wheat Pasta Sheets Partially Substituted with Pea Protein

**DOI:** 10.3390/foods13233798

**Published:** 2024-11-26

**Authors:** Chengyi Yang, Sze Ying Leong, Jessie King, Esther H.-J. Kim, Marco P. Morgenstern, Mei Peng, Dominic Agyei, Kevin Sutton, Indrawati Oey

**Affiliations:** 1 Department of Food Science, University of Otago, Dunedin 9054, New Zealand; yange860@student.otago.ac.nz (C.Y.); sze.leong@otago.ac.nz (S.Y.L.); jessie.king@otago.ac.nz (J.K.); mei.peng@otago.ac.nz (M.P.); dominic.agyei@otago.ac.nz (D.A.); 2 Riddet Institute, Palmerston North 4442, New Zealand; esther.kim@plantandfood.co.nz (E.H.-J.K.); marco.morgenstern@plantandfood.co.nz (M.P.M.); kevin.sutton@plantandfood.co.nz (K.S.); 3 The New Zealand Institute for Plant & Food Research Limited, Lincoln 7608, New Zealand

**Keywords:** plant protein, vegan pasta, texture profile, mastication, oral processing

## Abstract

Plant proteins are increasingly incorporated into food products to enhance their nutritional value. However, little is known about how this alters the textural perceptions of such products. This study investigated the substitution of up to 35% wheat flour with pea protein isolate (PPI) into pasta sheets to determine how this influenced texture. Furthermore, an in vivo human mastication test (*n* = 116 participants) was conducted to evaluate oral tactile perceptions (perceived firmness, stickiness, and brittleness) and chewing time associated with PPI-containing pasta. Cooked pasta hardness decreased from 145 to 96 N at 5% PPI substitution due to the disruption of gluten network but increased to 144 N at 15–25% PPI substitution, indicating a stronger protein network at higher PPI substitution levels. In vivo, pasta substituted with 25% PPI required a shorter chewing time and was perceived as less firm, less sticky, and more brittle than wheat flour-only pasta. Regardless of pasta samples, fast chewers (average chewing time ≤13 s) were better at recognizing differences in pasta firmness, while slow chewers (>13 s) were more sensitive to changes in stickiness and brittleness. The results obtained in this study could contribute to the design of protein-rich pasta tailored to populations with specific texture requirements (e.g., softer texture for the elderly).

## 1. Introduction

There is a growing interest in incorporating plant proteins into foods to increase their nutritional value. Plant proteins derived from legumes are usually considered an ideal plant-based source due to their high production capacity and favorable amino acid scores [1]. Among the numerous legume proteins available, pea protein is one of the most popular due to its low allergenicity, greater digestibility, and complete amino acid profile [2,3]. However, different levels of incorporation can alter the protein network in a food, which in turn may influence the resulting physicochemical qualities, such as oral tactile perceptions, and nutritional value [4]. Therefore, it is important to understand how varying levels of incorporation of plant proteins impact the physicochemical properties of foods to ensure the quality of the final food products is not substantially compromised.

Pasta is one of the most common and popular staple foods worldwide and is generally made from semolina (durum wheat) or wheat (common wheat) flour, eggs, and water [5,6]. During the production of conventional pasta, wheat gluten plays a key role in forming fibril structures, which are crucial for determining the texture properties such as firmness, springiness, and chewiness, characteristics that are unique to pasta [7]. However, semolina is lower in protein content (13%) than plant-extracted proteins such as pea protein isolate (81%) [8,9]. Moreover, gluten is less digestible than other common protein sources. Based on the Digestible Indispensable Amino Acid Score (DIAAS), which is the current standard for assessing protein digestibility as endorsed by the Food and Agriculture Organization of the United Nations (FAO), gluten scores significantly lower at 0.25 compared to pea protein (0.88), as determined through studies in rats [10]. Therefore, incorporating pea proteins could be an effective method to enhance both the protein content and quality (e.g., physicochemical, textural, and sensory properties) in the resulting pasta [3,11]. Moreover, as a non-allergenic alternative to soy or dairy proteins, the inclusion of pea protein can broaden the appeal of flour-based products for consumers with dietary restrictions [3].

Pea proteins, derived from yellow peas (*Pisum sativum*), mainly composed of soluble proteins such as albumins and globulins, differ from wheat proteins, which consist of water-soluble albumins, salt-soluble globulins, alcohol-soluble prolamins (gliadins), and acid-soluble glutenins [12]. With a high protein content (>80%) and essential amino acids such as lysine, pea protein enhances the nutritional profile of flour-based products while addressing protein deficiencies in cereal-based flours. Functionally, such differences in protein composition and solubility can influence their water absorption capacity, potentially affecting the moisture retention of the resulting pasta during cooking. The absence of glutenins and gliadins (gluten complex) in pea protein, which typically provide strength and elasticity in wheat pasta dough [13], is expected to modify the viscoelasticity of the dough during cooking. Therefore, a complete replacement of wheat with pea protein in pasta dough is particularly challenging due to the lack of gluten-forming proteins in pea protein. In this context, a partial substitution approach is worth investigating to evaluate the potential impact of interactions between pea protein and wheat soluble proteins on the physicochemical and texture properties of cooked pasta as well as their effects on starch–protein binding, structural integrity, and oral tactile perceptions. A study by Laleg et al. [14] reported decreases in strong-linked (disulfide-linked or covalently linked) proteins and increases in weakly linked proteins when faba bean flour was incorporated into vegan pasta, highlighting that the interactions between wheat soluble proteins and legume proteins cannot be overlooked. While numerous studies have examined the incorporation of plant proteins into various foods including pasta [15,16,17], there remains a significant research gap regarding how the distinct functional characteristics and interactions between wheat and non-wheat proteins (such as pea protein in this context) influence the quality attributes (particularly texture) of cooked pasta as a function of cooking time.

Wheat pasta is characterized by its high cohesiveness and chewy oral tactile characteristics when consumed, thus requiring more intense mastication to break down its starch–gluten matrix before swallowing [18]. Despite this, the potential for improving the chewing characteristics of pasta through the incorporation of non-wheat proteins (such as pea protein), and by adjusting cooking methods of pasta (such as steaming), has not been widely explored, even though these factors are expected to influence the texture properties of pasta. In fact, multiple studies have revealed that human chewing behavior is greatly influenced by food texture [19,20]; for example, dry and hard food products usually require a longer chewing time for swallowing compared to foods with higher moisture content and lower hardness [21,22]. However, most studies in the current literature focus on natural foods or making comparisons between different food types, which inherently vary in composition. Such variability makes it challenging to obtain robust or consistent data on their texture properties, how their texture is being perceived, and how texture affects human chewing behavior, potentially introducing unexpected errors. To date, limited studies have explored the influence of food texture on human chewing behavior using defined food models with known ingredient ratios and hence controlled compositions, which could provide more reliable insights. Since humans exhibit varied chewing behavior from one individual to another, with chewing time being a key parameter that can influence their responses and ability to detect differences in oral tactile perceptions for foods with different texture properties, this area of research remains limited and may be of interest when incorporating pea protein into pasta.

Therefore, the main objective of this study was to investigate the impacts of pea protein incorporation on the textural properties of vegan pasta, using both instrumental and sensory techniques. To achieve this objective, firstly, the effect of partial substitution of wheat flour with pea protein isolate (up to 35% PPI substitution level) on the texture profiles (hardness, cohesiveness, springiness, and chewiness) of cooked pasta was followed as a function of cooking time (up to 30 min). Secondly, the oral tactile perception (perceived firmness, stickiness, and brittleness) of the cooked pasta with and without PPI was rated using 116 human participants and then compared on the basis of their chewing duration. The novelty of this study lies in two key aspects. First, unlike most previous research, the present study examines the texture profile of cooked pasta over varying cooking times rather than focusing solely on the optimum cooking time. Second, this study incorporates PPI to create pasta of distinct texture profiles, which was developed as a defined food model to investigate how human chewing duration affects their ability to detect textural differences during mastication.

## 2. Materials and Methods

### 2.1. Materials

A single batch of pea protein isolate (PPI, crude protein content = 85%, Peazazz^®^, Winnipeg, MB, Canada) was purchased through Caldic B.V. (Auckland, New Zealand). Plain wheat flour (WF, crude protein content = 11.5%, Woolworths) in bulk form was purchased from a local supermarket (Dunedin, New Zealand).

### 2.2. Preparation of Vegan Pasta Sheets Partially Substituted with Pea Protein Isolate

The formulations used for preparing the vegan pasta in this study are presented in Table 1. The water-to-dry ingredients (WF and PPI) ratio was fixed at 3:5 (*w*/*w*) for all formulations. Partial substitution of PPI with WF was conducted at mass percentages of 0, 5, 15, 25, and 35% for the dry ingredients, referred to as PPI0 (control), PPI5, PPI15, PPI25, and PPI35, respectively (Table 1). PPI substitution levels above 35% were evaluated in a preliminary study, but these formulations failed to produce consistently sized pasta sheets that remained intact without breaking (Supplementary Materials, Figure S1C,D). Additionally, the thickness of vegan pasta sheet was optimized at 2.1 mm during the preliminary study (Supplementary Materials, Figure S1A).

All vegan pasta sheets were produced using a pasta machine (Fimar MPF15N, Rimini, Italy) fitted with an MPF1.5 pasta Sfoglia shape disc. The pasta sheet preparation procedures were developed according to the Fimar user manual with modifications. Firstly, all dry ingredients were mixed using a Kenwood Mixer (KWL90.004SI, Hampshire, UK) at a speed level of 1 for 5 min. Next, the dry ingredients were transferred to the mixing chamber of the Fimar pasta machine, and distilled water was added. The dry ingredients and water were mixed, and the dough was kneaded for 8 min. Afterward, the pasta dough samples were passed through the Sfoglia shape disc fitted in the Fimar pasta machine to form sheets. The gap of the extrusion disc was adjusted to allow a constant thickness of pasta sheets (2.1 mm). Then, each pasta sheet was cut into a standard size of 150 × 135 mm and placed on baking paper to avoid sticking together. A total of 3 batches (3 × 1600 g) were produced for each formulation. Pasta sheets with baking paper were sealed inside a large plastic bag (680 × 395 mm) and stored at −18 °C until use within four weeks.

### 2.3. Pasta Cooking

Frozen pasta sheets were removed from the plastic bags and thawed at 20 °C for 30 min. Afterward, circular-shaped pasta samples were cut out from each sheet using a round-shaped cookie cutter (d = 20 mm). The sampling was conducted on three randomly selected sheets, with circular-shaped samples (“coins”) cut from the center of each sheet to avoid using potentially dried samples from the edges. For the in vivo mastication analysis of oral tactile perceptions, a maximum of 20 coins were sampled from a single pasta sheet (see Section 2.7).

The pasta coins were placed on a muffin tray (Vogue U.S., 12 cups, 90 mL per cup), with one pasta coin per muffin cup to allow sufficient hydration of pasta during cooking. The tray was then placed on a larger oven tray (530 × 325 mm), and boiling distilled water was added to the muffin tray to fill all cups (90 mL each), as well as to the larger oven tray. The tray was immediately loaded into a combi oven (Convotherm Maxx Pro, Eglfing, Germany) preheated to 100 °C, and the pasta was cooked with steam in the oven for either 5, 10, 15, 20, 25, or 30 min. The steam was added to maintain the oven temperature at 100 °C during the cooking process. Once the target cooking duration was reached, the tray was unloaded from the oven, and the pasta coins were transferred to a sieve, laid out independently, and drained for 5 min at 20 °C (room temperature). Afterward, each pasta coin was transferred to a lidded plastic container (20 mL, Bonson, Auckland, New Zealand) until analysis. The optimum cooking time for pasta was determined using AACC Method 66–50 (Supplementary Materials, Figure S2).

### 2.4. Determination of Moisture and Protein Content of Uncooked and Cooked Vegan Pasta

The moisture content of uncooked and cooked pasta coins was measured using the oven-drying method according to the work of Ahn et al. [23], with slight modifications. Briefly, moisture content was determined by calculating the weight loss on drying (LOD) in a hot-air oven (LabServ^®^ Overlay 27, Contherm Scientific Ltd., Wellington, New Zealand) at 100 °C for 24 h, with the assumption that 100% of LOD is due to water loss. The analysis of uncooked and cooked pasta coins was conducted on the same day of cooking.

The crude protein content of uncooked and cooked pasta coins was determined using the Kjeldahl method according to AACC Method 46-10.01, using a nitrogen-to-protein conversion factor of 6.25. All samples were analyzed in triplicate.

### 2.5. Texture Profile Analysis (TPA) of Cooked Vegan Pasta

To obtain consistent and representative TPA results, 15 pasta coins (pooled from 3 pasta batches, 5 coins from each batch) were analyzed for each PPI level (PPI0, 5, 15, 25, and 35%) and cooking time (5, 10, 15, 20, 25 and 30 min). The TPA analysis on pasta coins was adapted from the work of Wang et al. [24] with modifications. In this study, a double-compression test was conducted using a texture analyzer (TA.HD, Stable Micro Systems, London, UK) with a P/35 (d = 35 mm) cylinder probe and a 250 kg capacity load cell. Each sample was compressed twice with a 50% strain at the speed of 1 mm/s with 1 mm/s pre-test speed and 5 mm/s post-test speed. The gap time between the two compressions was 10 s. Hardness, springiness, cohesiveness, and chewiness, the texture profiles generally considered most applicable to human mastication, were calculated using Exponent (version 6.1.16.0, Stable Micro Systems, London, UK).

### 2.6. Microscopic Observation of Uncooked and Cooked Vegan Pasta

The microstructures of PPI0, PPI5, PPI15, PPI25, and PPI35 were observed before and after cooking for 20 min. Several pasta sections (thickness at 10 μm) were sampled from the frozen (−18 °C) pasta coins using a razor blade, mounted onto glass cavity slides (Fisher Scientific, Hampton, VA, USA), then stained with a water solution consisting of fluorescein sodium salt (0.05% *w*/*v*, Sigma Aldrich, St. Louis, MA, USA) to visualize starch and Rhodamine B (0.05% *w*/*v*, Sigma Aldrich, St. Louis, MA, USA) to visualize protein. After 30 min, the extra staining solution was removed using Kimwipes (Kimtech, Roswell, NM, USA), and the sample slides were sealed using a coverslip and nail varnish to avoid evaporation. Specimens were viewed using confocal laser scanning microscopy (Leica TCS SP5, Heidelberg, Germany) with the 20× objective. A fluorescein isothiocyanate and a tetramethyl rhodamine isothiocyanate filter block in the scanner were used for the excitation of dyes at wavelengths 488 and 561 nm, respectively. At least nine microscopy images from three sample section replicates were taken for pasta samples at each PPI level.

### 2.7. In Vivo Mastication Study of Cooked Vegan Pasta Evaluating Their Oral Tactile Perceptions and Chewing Duration

The in vivo mastication study was approved by the University of Otago Human Ethics Committee (Approval Reference Code: 23/092) and participants were informed in detail about the objectives and methodology of the study before signing a consent form. A total of 116 participants (36 male, 80 female), aged between 18 and 65 years old, were recruited from among university students and staff. Participants were required to complete a screening questionnaire to avoid including those with allergies, chewing problems, or those currently taking medications. To avoid the influences of starvation and food intake on the results, the in vivo study was not conducted during lunchtime (11:00 AM to 1:30 PM). To avoid chewing fatigue, only two types of pasta samples with similar cooking duration exhibiting comparable TPA profiles (e.g., hardness and cohesiveness, Section 2.5) were selected to be masticated by the human participants. Each pasta sample was coded with a random 3-digit code to prevent participants from knowing the sample identities. Cooking beyond 20 min weakened the pasta structure (see Supplementary Materials, Figure S3) and induced clear reductions in cohesiveness, springiness, and chewiness of PPI25 and PPI35 (Section 3.3 and Supplementary Materials, Figure S4). PPI25 and PPI0 pasta coins cooked for 20 min (Section 3.1) were chosen for the in vivo mastication study to determine whether an increased protein content due to PPI substitution in PPI25 would alter oral tactile perceptions compared to that of PPI0.

#### 2.7.1. Microbiological Testing of the Cooked Vegan Pasta Prior to In Vivo Mastication Study

Before in vivo analysis, a microbiological test was conducted on cooked pasta samples using the standard diluting and plating techniques to determine the number of colony-forming units (CFU) of pathogenic bacteria to ensure they met the microbiological limits for food (FSANZ-Standard 1.6.1). A plate count agar (PCA) was used to determine the total viable bacteria; a blood agar plate was adopted to determine the presence of *Staphylococcus*, *Streptococcus*, *Enterococcus*, and *Escherichia coli*; Oxford agar was used to detect the presence of *Listeria monocytogenes*, and Salmonella Shigella Agar (SS agar) to identify *Salmonella*. According to the standard, total variable bacteria in ready-to-eat food should be no more than 10^5^ CFU/g, and *Escherichia coli*, *Listeria*, and *Salmonella* should not be present.

A ten-fold dilution (10^−1^) was first conducted on the cooked pasta using a phosphate-buffered saline (PBS) solution. Afterward, 2 g of pasta sample was accurately weighed using a sterilized razor blade (boiled in water for 10 min then cooled down) on a Petri dish, then diluted with PBS solution in a sterilized stomacher sampling bag (7.5 cm × 18 cm, Whirl-Pak^®^, Pleasant Prairie, WI, USA). The sample with PBS was then mixed for 5 min using a stomacher (FER0400/002, Seward, West Sussex, UK). For PCA agar count, ten-fold serial dilutions were further conducted three times with PBS solution until the dilution series reached 10^−4^.

Spread plate technique was used to determine the CFU of pathogenic bacteria. To start, 0.1 mL of diluted sample suspension (10^−3^ and 10^−4^ for PCA, 10^−1^ for other agar plates) was transferred to the agar surface. A sterilized disposable spreading rod (Fisherbrand™, Hampton, VA, USA) was used to spread out the suspension on different agar types. For each sample, three replicates were conducted. The PCA plates were incubated aerobically for 72 h at 30 °C, and the Oxford and SS agar were incubated aerobically at 37 °C for 24 h. The blood agar plates were transferred into an anaerobic jar covered with an anaerobic bag (Mitsubishi Gas Chemical, Tokyo, Japan) and incubated at 37 °C for 24 h.

The numbers of colonies formed were counted and the results were calculated using Equation (1). The average of 30 replicates (10 sample replicates × 3 dilution replicates) was calculated as the final colony-forming units (CFU) per gram of pasta sample.

(1) $$\mathrm{CFU}\;\mathrm{per}\;\mathrm{gram}=\mathrm{number}\;\mathrm{of}\;\mathrm{colonies}\;\times \;\frac{1}{\mathrm{volume}\;\mathrm{plated}\;(\mathrm{mL})}\;\times \;\mathrm{dilution}\;\mathrm{factor}$$

where volume plated = 0.1 mL, and the dilution factor is the inverse of the total dilution being plated.

#### 2.7.2. Rating on the Oral Tactile Attributes of Cooked Vegan Pasta

Three oral tactile attributes associated with cooked vegan pasta: firmness, brittleness, and stickiness, were rated in the in vivo mastication study. Prior to evaluation, participants were provided with the definition for each attribute and trained to rate the attributes at the defined time points of chewing:

- Firmness is defined as the force required to bite completely through the sample between molars, which is rated at the first bite.
- Brittleness is the easiness of pasta to fall apart upon the application of a relatively small amount of force by teeth, which is rated at the first bite.
- Stickiness is the amount of pasta remaining in the molar teeth after chewing, which is rated before swallowing.

Participants were trained to use the line scales for rating. They were also trained to chew the samples using only their molar teeth to standardize the chewing process. Since slight differences in color between two cooked samples were observed (Supplementary Materials, Table S1), the individual tasting booth was used, and samples were served one by one after randomization to eliminate order effects. Each booth was equipped with a computer with the questionnaire loaded, one tray with a plastic container (containing one pasta coin), one plastic spoon, a spitting container, a digital timer, a napkin, and a glass of filtered drinking water (see Supplementary Materials, Figure S5). Participants were asked to consume 1/3 of the pasta coin at a time and rate each oral tactile attribute (firmness, brittleness, and stickiness) on a 150 mm line scale, where the leftmost end of the scale was labeled “Not firm at all”/“Not brittle at all”/“Not sticky at all”, while the rightmost end was labeled “Extremely firm”/“Extremely brittle”/“Extremely sticky”.

A one-minute rest was given after participants finished masticating the first sample and then the subsequent samples were provided, with the order of PPI0 and PPI25 randomized. The presentation order of samples followed a William Square Design, and the questionnaire design was conducted using Compusense Online (Compusense Inc., Guelph, ON, Canada). The results were recorded as the distance (mm) from the original point (0) to the point marked by participants. The distance (mm) was then normalized into a percentage (%) using Equation (2). Data from four participants who did not complete the study were excluded from the analysis.

(2) $$\mathrm{Normalized}\;\mathrm{firmness}/\mathrm{brittleness}/\mathrm{stickiness}=\frac{\mathrm{Distance}\;\mathrm{from}\;\mathrm{the}\;\mathrm{original}\;\mathrm{point}\;\left(\mathrm{mm}\right)}{150}\;\times \;100\%$$

#### 2.7.3. Chewing Time Determination for Cooked Vegan Pasta

In the same sensory evaluation, participants were provided with two samples (PPI0 and PPI25 in randomized order, one coin (2 g) for each sample, and a one-minute break in between samples) and a digital timer to help them record their chewing time for each sample. The participants were informed that the duration of time from the first bite to the time point that the sample was able to be swallowed was defined as chewing time. The participants were asked to either swallow the samples or expectorate samples into the spitting container. The expectorated samples were then disposed of through a bio-safety disposal process. Participants were given a cup of filtered drinking water (250 mL) for rinsing their mouths and refreshing their palates in between samples. The participants whose average chewing time of both samples was shorter than the median average chewing time of all participants (*n* = 116) were categorized as fast chewers; those with chewing times longer than the median were categorized as slow chewers. The rating differences in the oral tactile perceptions (from Section 2.7.2) between the two chewing groups were further evaluated.

### 2.8. Statistical Analysis

Statistical analysis was performed using SPSS (version 29, IBM Corp., Chicago, IL, USA) and R (version 4.3.1, Boston, MA, USA). The normally distributed data were presented as the mean ± standard deviation (SD), while the non-normally distributed data (e.g., rating of oral tactile perceptions) were presented using violin plots with boxes labeled with first quartile (Q1), median, and third quartile (Q3). Statistical significance was defined as a *p*-value lower than 0.05. For moisture and protein contents and texture properties, a two-way analysis of variance (ANOVA) with Tukey’s HSD test was conducted, considering PPI levels and cooking time as factors. To compare the significance of moisture and protein content before and after cooking, an independent t-test was conducted. A paired t-test was used for in vivo mastication ratings of oral tactile attributes and chewing time between PPI0 and PPI25 samples. To compare the oral tactile perceptions of PPI0 and PPI25 samples between fast and slow chewers (categorized based on their chewing time), an independent t-test was conducted.

## 3. Results and Discussion

### 3.1. Effect of Partial Substitution of PPI on the Physicochemical Properties and Microstructure of Uncooked and Cooked Pasta

**Figure 1:**
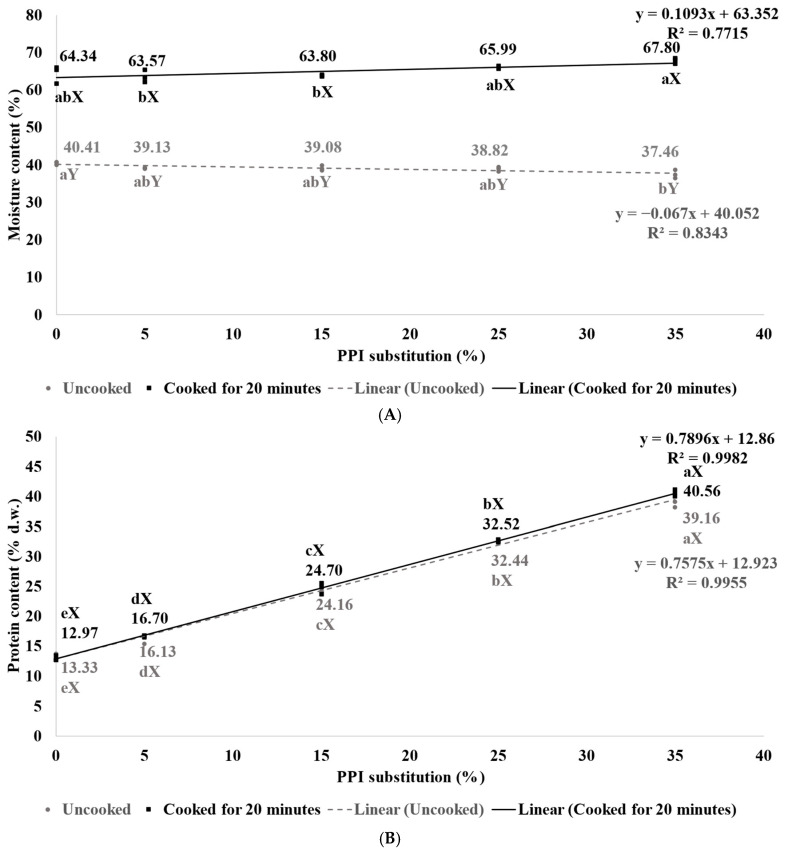
Moisture (**A**) and protein (**B**) content (*n* = 3) of uncooked and cooked (20 min) vegan pasta samples with PPI substitution levels up to 35%. Mean values with different lowercase letters indicate a significant difference (*p* < 0.05) in the moisture or protein content between vegan pasta made with varying PPI levels, when cooked for the same duration. Mean values with different uppercase letters indicate a significant difference (*p* < 0.05) in the moisture or protein content between vegan pasta made with the same PPI level but cooked for different durations. The dashed line (- - -) represents the trend of uncooked pasta with PPI substitution, and the solid long line (––––) represents the trend of cooked (20 min) pasta with PPI substitution. d.w.: dry weight.

The moisture and protein content of pasta produced in the present study were determined before and after 20 min of cooking (Figure 1), as previous studies indicated that texture profiles and perceptions of cooked pasta could be greatly influenced by the moisture and protein content [25,26,27,28]. The moisture content of uncooked pasta exhibited a decreasing trend, albeit quite mildly, with increased PPI substitution (Figure 1A), indicating the PPI-containing pasta was slightly more susceptible to moisture loss. The globular alkali-extracted PPI has been shown to exhibit a lower water-holding capacity (WHC = 2.4–2.6 g/g) compared to fibril wheat gluten (2.8 g/g) [29,30]. Therefore, PPI0, with its strong gluten network, is expected to better hold the moisture during freezing and storage than weak globular pea protein structures. However, after cooking, an increase in moisture content was observed for pasta samples substituted with PPI up to 15%. With further PPI substitution levels, the moisture content increase in these pasta samples after cooking was even greater (27% for PPI25 and 30% for PPI35). A study by Webb et al. [31] revealed that the water absorption capacity of PPI (ranging between 2.7 and 3.8 g/g) was almost two times higher than that of vital wheat gluten (1.4 g/g). This is because pea proteins are primarily composed of soluble proteins such as albumins and globulins, in contrast to wheat proteins, which include water-soluble albumins, salt-soluble globulins, alcohol-soluble prolamins (gliadins), and acid-soluble glutenins, thus leading to the more hydrophobic nature of wheat gluten [32]. Gluten is a polymer protein network formed by the crosslinking of gliadin and glutenin connected by strong interchain covalent (disulfide) bonds in the presence of water [33]. When wheat is partially substituted with pea protein, the globulins in pea protein can disrupt these disulfide bonds within the gluten network, potentially breaking the links between gliadin and glutenin [34]. The weakened protein network could then allow greater access to water and enhance water retention during cooking, thus explaining the observed increase in moisture content in pasta samples substituted with PPI up to 15%.

An increasing trend of the protein content (13% d.w. to 40% d.w.) was observed with increased PPI substitution from 0 to 35%, respectively. However, this trend did not change with cooking (Figure 1B), indicating minimal leaching of starch or protein, regardless of the protein source (WF or PPI). Teterycz et al. [15] investigated the physicochemical properties of pasta incorporating 0–20% green pea, red lentil, and grass pea flours, reporting a protein content of 12.4% d.w. for the conventional semolina durum pasta (control pasta), while legume-incorporated pasta demonstrated a higher protein content of approximately 40% d.w. A significant increase in protein content of the legume flour-incorporated pasta was also reported by Wójtowicz and Mościcki [16], who investigated the physicochemical properties of pasta with white bean, yellow pea, and lentil flour incorporated. In the present study, pasta cooked for up to 20 min did not induce a significant loss of protein, which was unexpected since previous studies revealed a significant reduction in protein content of legume-incorporated pasta after cooking, corresponding to high cooking loss [15,16,35,36]. This outcome is attributed to the unique cooking approach used in this study (i.e., oven cooking with steam in 100 °C preheated water) instead of conventional boiling in the pot, as adopted by previous studies. The oven cooking method involving steam minimized cooking losses and enabled the comparison of pasta texture at a consistent weight.

**Figure 2:**
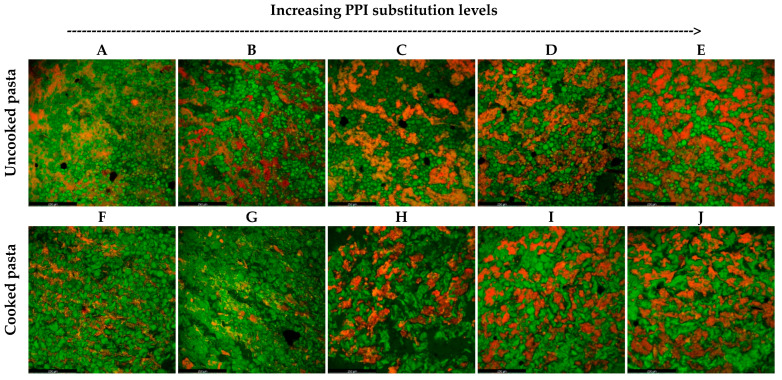
Confocal laser scanning microscopy images (CLSM, magnification 20×, scale bar 250 µm) of the cross-sectional view of uncooked and cooked (20 min) pasta: uncooked PPI0 (**A**); uncooked PPI5 (**B**); uncooked PPI15 (**C**); uncooked PPI25 (**D**); uncooked PPI35 (**E**); cooked PPI0 (**F**); cooked PPI5 (**G**); cooked PPI15 (**H**); cooked PPI25 (**I**); cooked PPI35 (**J**); Protein stained with rhodamine B (red); starch stained with fluorescein sodium salt (green).

The morphology of the uncooked and cooked (20 min) pasta samples was analyzed using confocal laser scanning microscopy (CLSM) (Figure 2), which provided an optical cross-sectional view of pasta samples through 3-D specimen images. A typical pasta structure was observed for PPI0 samples, with starch granules (green) embedded in the long fibrous gluten network (red) (Figure 2A), similarly reported by Sissons et al. [37]. As expected, a higher amount of protein (red stain) was observed with the increased levels of PPI substitution (Figure 2B–E). However, the protein structure (red stain structure) gradually changed in appearance from being a long fibrous network to being denser but with less organized “clumps”. The clumps resulted from the accumulation of globular PPI during pasta production. At PPI5, both a long fibrous gluten network and globular PPI clumps were clearly observed (Figure 2B). Clearly, the presence of PPI clumps diluted the fibrous structure (compared to PPI0) and hindered the formation of a more continuous gluten network.

The microstructure of pasta was also influenced by cooking (Figure 2A–E vs. Figure 2F–J); after 20 min, starch swelling and gelatinization were observed, with cooked pasta samples containing higher levels of PPI showing greater degrees of gelatinization (Figure 2G–J). This was likely due to the disruption of the gluten network. Less protein network enveloping the starch granules suggests a higher chance of water gaining access to starch granules during cooking. It is important to note that no clear morphology changes in protein were observed before and after cooking of any PPI-containing pasta.

### 3.2. Effect of Partial Substitution of PPI on the TPA Texture Properties of Cooked Pasta

TPA with a double-compression test was conducted on PPI-incorporated pasta samples cooked for different durations to evaluate whether PPI substitution altered their texture profiles, including hardness, cohesiveness, springiness, and chewiness. ANOVA analysis (Table 2) revealed that all measured texture properties were significantly (*p* < 0.001) influenced by both the PPI substitution level and the cooking time. Of these texture properties, springiness was the least affected, with a lower F-value (8.177) compared to other texture properties (F-values ranging from 16 to 46). The springiness refers to the degree to which food reverts to its original state when the force is removed [38]. The springiness of pasta with low PPI levels (PPI0–5) was not greatly influenced by cooking time, which likely explained the low F-value. An et al. [39] mentioned that the 3-D fibrillar structure of gluten was essential for providing the springiness of pasta. It can be postulated that the pasta with low PPI levels still retained a higher proportion of the extended 3-D gluten network, helping to maintain its springiness during cooking (Figure 2F,G).

Extending the cooking duration from 5 to 30 min was found to reduce both the hardness and chewiness of PPI0 without affecting their cohesiveness or springiness (Table 3). The effect of prolonged cooking time on the reduction in hardness and chewiness in vegan pasta was also observed in samples with PPI substitution. A longer cooking time increases the moisture content of cooked pasta (Supplementary Materials, Figure S6), which has been shown to lead to a decrease in pasta hardness [40]. Chewiness (hardness × cohesiveness × springiness) is the energy required to chew the food until it is ready to be swallowed and has been reported to positively correlate with hardness [41,42]. Therefore, it was not surprising to observe a reduction in chewiness alongside hardness as cooking time was extended. Unlike PPI0, the PPI-substituted pasta showed decreases in both cohesiveness and springiness when cooked beyond 20 min, with their reductions becoming more pronounced at higher levels of PPI substitution. This was likely due to the breakage of the pasta during the first compression of TPA. Cohesiveness refers to the retention capacity of the sample against deformation upon the force applied [43]. It can be postulated that the overcooking of pasta beyond 20 min induced breakage of its overall structure. Therefore, once force was applied, pasta samples with high PPI levels (PPI25 and PPI35) broke down and were unable to recover to their original shape, resulting in low cohesiveness and springiness (Supplementary Materials, Figure S3).

Compared to cooking time, PPI levels displayed a more significant influence on the texture properties of cooked pasta, with the ANOVA analysis indicating higher F-values (38 to 133) than those for cooking time (8 to 46) (Table 2). After 20 min of cooking, the decrease in hardness at a low level (5%) of PPI substitution (Table 3) may be attributed to the disruption of the gluten network (Section 3.1).

Similar reductions in the pasta firmness with the incorporation of legume flour into pasta at low levels (0–10%) have been reported by Teterycz et al. [15] and Zhao et al. [37]. Gluten networks, which are strongly stabilized by disulfide bonds, play a critical role in reinforcing pasta structure, contributing to its firmness [44]. However, the incorporation of legume proteins, such as faba flour as observed by Laleg et al. [45], into pasta can hinder gluten network formation, weakening the overall structural integrity and thus explaining the results observed in the current study. Laleg et al. [45] also reported an increased amount of sodium dodecyl sulfate (SDS)-soluble (weakly linked) protein for pasta with faba flour incorporated, accompanied by a decrease in dithioerythritol (DTE)-soluble (disulfide-linked, medium molecular weight) and non-extractable (covalently, other than disulfide-linked, higher molecular weight) proteins, further indicating a compromised structural strength with legume protein incorporation.

An increase in the hardness of 48 N at higher PPI levels (5–25%) of cooked pasta (20 min) (Table 3) can be explained by the higher protein content (32.52% d.w. for PPI25 vs. 16.70% d.w. for PPI5) (Figure 1B). Petitot et al. [25] found that the hardness of cooked pasta increased by 38% when 35% split pea flour was incorporated into durum flour pasta. Similarly, Zhao et al. [17] reported a decrease in firmness of cooked pasta at low levels (0–10%) of yellow pea flour incorporation but observed an increase in firmness when the incorporation level was raised to 10–30%. In the present study, a denser protein network was observed with the increase in PPI levels (Figure 2G–J), which might explain the increase in the hardness of cooked pasta. Sisson et al. [37] also observed a denser protein network of spaghetti with increased protein content, together with the increase in spaghetti firmness. Meanwhile, the study conducted by Islam et al. [46] reported a positive correlation between the degree of starch gelatinization (DSG) and the hardness of parboiled rice. Therefore, the increase in starch gelatinization as observed in Figure 2G–J could also explain the increased hardness of PPI-containing cooked pasta. In contrast, the decrease in hardness observed in PPI35 was likely due to the high moisture content (67.8%) of the cooked samples compared to lower PPI samples (63–65%) (Figure 1A).

When comparing pasta that was cooked for 20 min, the springiness and chewiness of cooked pasta were found to increase with higher levels of PPI substitution (from 5% to 35%). Since cohesiveness refers to the retention capacity of the sample against deformation under applied force [43], the cohesiveness of pasta decreased at lower levels of PPI substitution (5–15%) but increased as the PPI substitution level was further elevated (Table 3). This phenomenon can be explained by the disruption of the gluten network at lower PPI levels, leading to reductions in hardness, as well as the cohesiveness of the cooked pasta. However, as PPI levels increased further, the globular pea proteins began to replace wheat gluten, providing greater resistance to compression and subsequently increasing cohesiveness. The springiness and chewiness of PPI0 were consistently higher than in all PPI-containing cooked pasta samples. In PPI0 (made entirely from WF), a strong gluten network was formed, whereas, in PPI-containing pasta samples (PPI5–35), the presence of globular PPI diluted the gluten network, resulting in the formation of a pea-gluten network instead. According to An et al. [39], the fibril structure of gluten is essential for providing the springiness of pasta. However, the present study observed that the springiness of cooked pasta increased with increased levels of PPI (Table 3). This may be related to the higher moisture content of samples with greater levels of PPI substitution (PPI25 and PPI35) (Figure 1A), which may suggest a degree of lubrication and flexibility within the structure, thus enhancing springiness. Typically, the chewiness (hardness × cohesiveness × springiness) is positively related to the springiness [24]. Thus, it is not surprising to observe that the chewiness of high PPI samples (PPI25 and PPI35) increased along with springiness.

The TPA results indicated that low levels of PPI substitution may compromise the texture properties of cooked pasta, potentially exerting a significant influence on oral tactile perceptions during mastication by humans. In contrast, PPI substitution levels above 25% appeared to enhance some texture properties (e.g., hardness) to levels comparable to the control (PPI0), while also increasing protein content.

### 3.3. Effect of Partial Substitution of PPI on the In Vivo Oral Tactile Perceptions and Chewing Duration of Cooked Pasta

After 20 min of cooking, the TPA results showed that the hardness of cooked PPI0 was similar to that of PPI15 and PPI25; its cohesiveness was comparable to PPI5 and PPI25; and its springiness matched PPI35 (Table 3). These similarities suggest that PPI25 shared several TPA properties with PPI0, indicating that the pea–gluten network formed in PPI25 was comparable to the gluten network in PPI0. At the same time, the moisture content of PPI25 was found not to be significantly different from PPI0 (Figure 1A). However, during the preliminary sensory trial (*n* = 30) conducted as a pilot focus group for participants to describe the oral tactile perceptions of both samples, the main differences in chewing characteristics of pasta when PPI was incorporated were described as the loss of firm and cohesive texture, which was replaced by the granular and brittle one that was easily broken down and dispersed in the mouth after chewing. “Sticky” was mentioned by participants for PPI0 to describe the difficulty of removing the remaining pasta from teeth during chewing. Instead, “grainy” was more frequently reported for PPI25. It is important to note that both brittleness and stickiness are difficult to detect with the double-compression texture analysis (Supplementary Materials, Figure S3). Given that sensory perception often provides a more nuanced understanding of texture, a sensory test was conducted with human participants (*n* = 116) in the present study to masticate cooked samples of PPI0 and PPI25. Participants were trained specifically to rate the firmness, brittleness, and stickiness of pasta after being cooked for 20 min. The outcome of this sensory testing is expected to reflect the actual consumer experience of masticating cooked pasta and to assess how texture perceptions align or differ from instrumental results. Additionally, total chewing time for each cooked pasta sample was recorded to examine its potential influence on oral tactile perceptions, specifically for firmness, brittleness, and stickiness.

**Figure 3:**
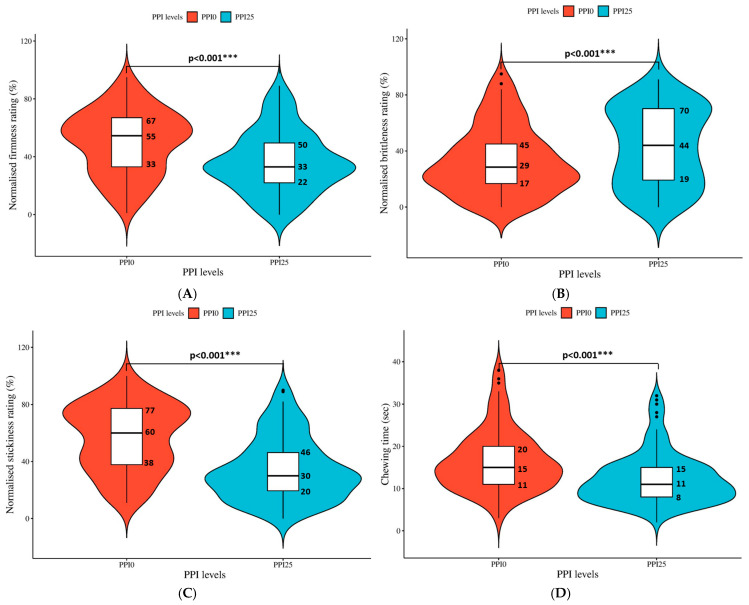
Distribution of participant ratings on perceived firmness (**A**); brittleness (**B**); stickiness (**C**); and chewing time (**D**) of two cooked pasta samples: 100% wheat flour (PPI0, red), and a blend of 75% wheat flour and 25% pea protein isolate (PPI25, blue). The width of the violin shape represents the frequency of the corresponding rating (or chewing time). The numbers beside each box plot indicate the first quartile (Q1), median, and third quartile (Q3) values (read from bottom to top). *** indicates a significant difference (*p* < 0.001) between the ratings (or chewing time) of PPI0 and PPI25.

The in vivo sensory testing revealed that the introduction of 25% PPI-containing cooked pasta significantly decreased (*p* < 0.001) the perceived firmness (average rating decreased from 50 to 36) and stickiness (average rating decreased from 57 to 34), while a slight increase in pasta brittleness (average rating increased from 33 to 43) was observed by participants (Figure 3A–C) compared to PPI0. A similar observation was noted by Susanna and Prabhasankar [47] involving 15 participants, who reported a lower perceived firmness of gluten-free pasta compared to the control (100% durum flour pasta). A study (*n* = 9) conducted by Khouryieh et al. [48] also reported a lower perceived firmness of noodles made from soy flour compared to the control (wheat flour).

The higher perceived firmness of PPI0 compared to PPI25 was unexpected, as the TPA results indicated no significant difference in hardness between them (Table 3). The different methods of evaluation of firmness might be responsible for the disagreement in results. TPA involves a double-compression test using a 35 mm diameter cylinder probe, while chewing involves the compression and shearing of pasta samples with the molar teeth. During the shearing procedure, the strong gluten network of PPI0 could have contributed to a higher perceived firmness of pasta samples. Clearly, the double-compression test conducted by the texture analyzer could not accurately reflect oral tactile perceptions. Likewise, Manthey and Dick [49] found that the compression-type probes were less sensitive in detecting the difference in the firmness of pasta made of different formulations than human panels.

Stickiness, as reflected by the participants, was another important perception determining their overall chewing time. Stickiness is related to the amount of amylose and amylopectin leached from the gelatinized starch granules during cooking [47] and is dependent on their total content [50]. The mechanism likely involves the bonding and interactions between amylose and amylopectin in the leachate, which leads to the formation of a highly sticky surface on the pasta [50]. PPI0 (made entirely from WF) was rated with higher perceived stickiness than PPI25 (Figure 3C), probably due to the higher starch content of WF (~70%) than PPI (not determined) [51], thus indicating a greater amount of amylose and amylopectin leaching during cooking. Other researchers have reported similar observations. For example, a lower perceived stickiness of gluten-free pasta made of soy flour was reported (*n* = 15) by Susanna and Prabhasankar [47].

The average normalized rating of perceived brittleness of PPI25 was 30% higher than that of PPI0 (Figure 3B). The low perceived brittleness of PPI0 might be attributed to its high gluten concentration. Compared to PPI25, CLSM analysis revealed that the strong and highly organized gluten network of PPI0 contributed strength to hold the structure of pasta, which increased the force required to break down the whole network (Figure 2F vs. Figure 2H).

The in vivo analysis also revealed that the average chewing time for PPI0 (16 s) was 33% longer than that of PPI25 (12 s) (Figure 3D), which could be due to the high perceived firmness and stickiness, as well as the low perceived brittleness of PPI0. The high firmness of pasta implies a greater effort was required to break it down, increasing the number and time of chewing cycles. The high stickiness of PPI0 pasta increased chewing time because participants spent more time getting rid of the sticky samples on their teeth, as reflected by them, while PPI25 pasta samples with high brittleness were easily broken down, thus less chewing cycles were required to achieve the particle size suitable for swallowing. Based on the results, it was postulated that the PPI incorporation could be a potential strategy to create pasta that is easier to chew, together with an improved protein content. With this knowledge, texture-modified foods could be developed tailored to specific groups. For example, the elderly often consume less dietary protein than the current recommended intake [52], usually require longer times of chewing than youths and adults [53], and prefer foods with soft textures that allow them to chew easily [54].

### 3.4. The Relationships Between Human Chewing Duration and Their Oral Tactile Perceptions for Cooked Pasta with and Without Partial Substitution with PPI

**Figure 4:**
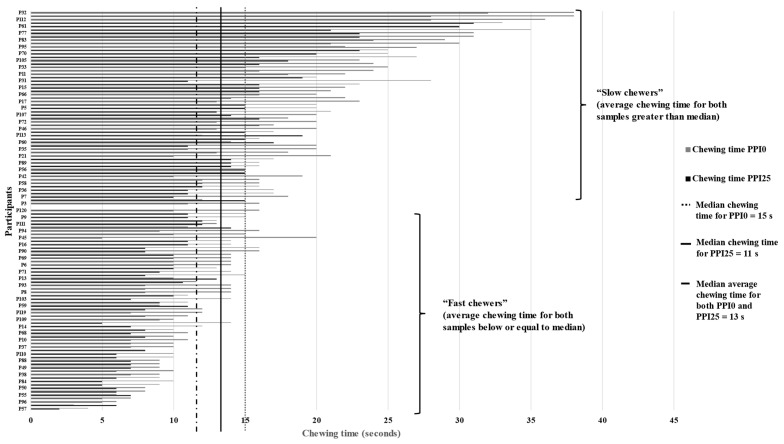
Variations in the chewing time of participants (*n* = 116) for PPI0 and PPI25. The dotted (······) and dash-dot (– · –) lines represent the median chewing time of PPI0 and PPI25, respectively. The solid long line (––––) represents the median average chewing time of both samples. Participants with average chewing time for both samples below or equal to the median value (13 s) were defined as “fast chewers”; and participants with average chewing time for both samples greater than the median value (13 s) were defined as “slow chewers”.

To further investigate the relationship between chewing time and oral tactile perceptions, participants were grouped based on their average chewing time for the two PPI samples. The calculated median (*n* = 116) average chewing time of both samples was 13 s. Thus, fast chewers (*n* = 59) were defined by an average chewing time (of both samples) below or equal to 13 s, while slow chewers (*n* = 57) were those with an average chewing time (of both samples) greater than 13 s (Figure 4).

Interestingly, it was found that both fast and slow chewers scored the firmness and stickiness of PPI0 significantly higher than PPI25 (*p* < 0.05) (Table 4). Fast chewers were more likely to rate firmness higher for PPI0 (average rating 51.7), and lower for PPI25 (35.3), with a higher t-value (t = 4.1, *p* < 0.001) than slow chewers (48.4 vs. 37.2 for PPI0 vs. PPI25, t = 2.8, *p* = 0.007). In contrast, stickiness was more likely to be rated lower for PPI25 by slower chewers (56.6 vs. 32.6 for PPI0 vs. PPI25, t = 4.9, *p* < 0.001) than fast chewers (56.5 vs. 35.9 for PPI0 vs. PPI25, t = 5.6, *p* < 0.001). This suggests that fast chewers were likely more sensitive in detecting differences in firmness, while slow chewers were better able to detect differences in stickiness. Given the average chewing time of fast chewers (t = 9.8 s) was shorter compared to slow chewers (t = 19.3 s), fast chewers may have found it easier to distinguish the differences in firmness, which is typically perceived at the initial stage of chewing [55]. On the other hand, stickiness refers to the interaction between samples and teeth, which is typically perceived during late chew-down [55]. Therefore, slow chewers engaging in longer chewing times could probably identify the differences in stickiness between the pasta samples more easily.

No significant difference in brittleness was perceived by fast chewers (t = −1.2, *p* = 0.225). However, slow chewers perceived PPI25 to be more brittle (t = −3.2, *p* = 0.002). It appeared that slow chewers were more capable of distinguishing the brittleness of pasta samples than fast chewers. This is likely attributed to the longer chewing time, which allows slow chewers to effectively perceive the gradual breakdown of the pasta during mastication. Liu et al. [18] provided a conceptual framework of oral processing considering the dominant sensory and physiological parameters of solid foods during oral processing. In the framework, hardness (firmness) was classified as the “food experience” that can be directly perceived by participants, while brittleness (“fracture”) and stickiness (“particle-saliva-oral cavity mechanics”) involved “interactions” to be perceived. Therefore, in the present study, it was postulated that fast chewers were more capable of distinguishing the direct “food experience”, such as firmness. Comparatively, slow chewers were more sensitive to those perceptions that require “interactions”, such as brittleness and stickiness. The insights gained from this study are valuable for designing foods with more appealing textures to improve the consumer experience for specific consumer groups. For example, it is recommended to prioritize texture attributes such as brittleness and stickiness when designing foods targeting elderly consumers, who are mostly slow chewers [53].

## 4. Conclusions

In this study, vegan pasta with PPI incorporation significantly altered the protein content and texture properties, as well as the oral tactile perceptions of cooked pasta. PPI incorporation could be a potential strategy to create pasta that is easier to chew while also exhibiting an improved protein content. The substitution of PPI at low levels (5–15%) led to a decrease in pasta hardness and cohesiveness, however, enhanced pasta hardness and cohesiveness were observed at higher levels of PPI substitution (15–25%). Additionally, PPI incorporation seemed to decrease the springiness and chewiness of pasta. With the increased levels of PPI substitution, the morphology of pasta samples presented a transition in the protein structure from one with a long fibrous network to one that was denser but with less organized “clumps”. The in vivo analysis revealed a decrease in chewing time when 25% PPI was substituted into pasta. At the same time, decreases in oral tactile perceptions of firmness and stickiness, together with an increase in brittleness, were observed. Furthermore, this study found differences in sensitivity to oral tactile perceptions between fast and slow chewers. Such findings could help design protein-rich foods with more appealing textures to improve the experience of specific consumer groups (e.g., the elderly who are mostly slow chewers). However, the in vivo analysis also revealed differences between results generated by the texture analyzer and humans. Effectively interpreting oral tactile perceptions using mechanical results remains a challenge, and this could serve as a key focus for future studies. The use of a mechanical chewing simulator, designed to mimic mastication and replicate oral functions, could be used in future research to verify agreements or disagreements between instrumentally measured texture properties and in vivo human oral tactile perceptions, comparing conventional and PPI-containing pasta. Additionally, other oral processing parameters, such as the number of chews and duration of one chewing cycle, could be examined when future studies are conducted on the chewing behavior of PPI-containing pasta. 

## Figures and Tables

**Table 1:** Formulation of pasta sheets at increasing levels of pea protein substitution.

Sample Code	PPI Substitution (*w*/*w*%)	Ingredients (g) per Batch			
		Wheat Flour (WF)	Pea Protein Isolate (PPI)	Distilled Water	Total Weight
PPI0	0%	1000	0	600	1600
PPI5	5%	950	50	600	1600
PPI15	15%	850	150	600	1600
PPI25	25%	750	250	600	1600
PPI35	35%	650	350	600	1600

**Table 2:** Summary of the one-way ANOVA results on the significant differences in the texture properties (TPA) of PPI-incorporated pasta after cooking according to PPI levels, cooking time, and PPI levels × cooking time (two-way interaction).

Variables	Interaction	F-Value	*p*-Value
Hardness	PPI levels	115.301	<0.001
	Cooking time	46.262	<0.001
	PPI levels × Cooking time	1.641	0.044
Cohesiveness	PPI levels	133.129	<0.001
	Cooking time	16.823	<0.001
	PPI levels × Cooking time	6.496	<0.001
Springiness	PPI levels	59.429	<0.001
	Cooking time	8.177	<0.001
	PPI levels × Cooking time	1.516	0.076
Chewiness	PPI levels	38.122	<0.001
	Cooking time	20.110	<0.001
	PPI levels × Cooking time	2.971	<0.001

**Table 3:** TPA texture properties of vegan pasta at different PPI substitution levels when cooked between 5 and 30 min.

TPA Texture Attribute (*n* = 15)	PPI Levels of Substitution (*w*/*w* %)				
	0% (Control)	5%	15%	25%	35%
Cooking time = 5 min					
Hardness	168.50 ± 24.75 ^a^_W_	134.19 ± 20.10 ^b^_W_	159.00 ± 19.78 ^a^_W_	168.49 ± 16.09 ^a^_W_	134.64 ± 21.55 ^b^_W_
Cohesiveness	0.45 ± 0.10 ^b^_W_	0.48 ± 0.05 ^b^_W_	0.32 ± 0.06 ^c^_W_	0.5 ± 0.06 ^b^_W_	0.67 ± 0.06 ^a^_W_
Springiness	0.69 ± 0.13 ^a^_W_	0.48 ± 0.08 ^b^_W_	0.51 ± 0.20 ^ab^_W_	0.65 ± 0.07 ^a^_W_	0.66 ± 0.06 ^a^_WX_
Chewiness	51.21 ± 15.18 ^a^_WX_	30.63 ± 6.09 ^b^_W_	27.53 ± 15.76 ^b^_W_	55.11 ± 11.19 ^a^_W_	59.53 ± 13.54 ^a^_W_
Cooking time = 10 min					
Hardness	165.74 ± 21.12 ^a^_W_	117.64 ± 19.45 ^b^_WX_	163.34 ± 20.15 ^a^_W_	170.54 ± 12.51 ^a^_W_	118.48 ± 19.72 ^b^_WX_
Cohesiveness	0.49 ± 0.09 ^b^_W_	0.46 ± 0.07 ^b^_WX_	0.34 ± 0.06 ^c^_W_	0.45 ± 0.05 ^b^_W_	0.73 ± 0.06 ^a^_W_
Springiness	0.76 ± 0.14 ^a^_W_	0.45 ± 0.08 ^c^_W_	0.51 ± 0.16 ^c^_W_	0.61 ± 0.07 ^b^_WX_	0.60 ± 0.06 ^b^_XY_
Chewiness	63.73 ± 26.47 ^a^_W_	24.52 ± 6.54 ^c^_WX_	29.49 ± 14.93 ^bc^_W_	47.62 ± 11.86 ^ab^_WX_	51.95 ± 10.26 ^a^_WX_
Cooking time = 15 min					
Hardness	146.66 ± 10.11 ^b^_WX_	111.49 ± 18.68 ^c^_WXY_	170.59 ± 15.98 ^a^_W_	150.21 ± 17.35 ^b^_WX_	103.73 ± 9.42 ^c^_X_
Cohesiveness	0.49 ± 0.10 ^b^_W_	0.47 ± 0.04 ^b^_WX_	0.35 ± 0.04 ^c^_W_	0.49 ± 0.07 ^b^_WX_	0.69 ± 0.04 ^a^_W_
Springiness	0.75 ± 0.15 ^a^_W_	0.47 ± 0.05 ^c^_W_	0.52 ± 0.18 ^bc^_W_	0.63 ± 0.08 ^abc^_WX_	0.69 ± 0.08 ^ab^_W_
Chewiness	56.43 ± 22.89 ^a^_WX_	24.39 ± 3.51 ^b^_WX_	32.44 ± 15.76 ^ab^_W_	47.06 ± 16.37 ^a^_WX_	49.66 ± 7.68 ^a^_WX_
Cooking time = 20 min					
Hardness	145.22 ± 8.68 ^a^_WX_	96.44 ± 15.25 ^b^_XYZ_	143.63 ± 15.25 ^a^_WX_	144.55 ± 21.62 ^a^_X_	85.84 ± 7.41 ^b^_Y_
Cohesiveness	0.52 ± 0.08 ^b^_W_	0.46 ± 0.03 ^bc^_WX_	0.38 ± 0.06 ^c^_W_	0.47 ± 0.05 ^b^_WX_	0.69 ± 0.05 ^a^_W_
Springiness	0.80 ± 0.1 ^a^_W_	0.48 ± 0.06 ^c^_W_	0.58 ± 0.17 ^bc^_W_	0.61 ± 0.07 ^bc^_WX_	0.69 ± 0.05 ^ab^_WX_
Chewiness	60.37 ± 16.93 ^a^_WX_	20.83 ± 3.38 ^c^_X_	32.73 ± 14.30 ^bc^_W_	42.11 ± 14.05 ^b^_WX_	40.39 ± 3.80 ^b^_X_
Cooking time = 25 min					
Hardness	136.70 ± 23.55 ^a^_XY_	86.33 ± 13.69 ^b^_YZ_	133.12 ± 27.97 ^a^_X_	139.51 ± 14.63 ^a^_X_	75.36 ± 4.76 ^b^_Z_
Cohesiveness	0.47 ± 0.04 ^a^_W_	0.43 ± 0.03 ^ab^_WX_	0.37 ± 0.01 ^b^_W_	0.40 ± 0.05 ^b^_XY_	0.47 ± 0.10 ^a^_XW_
Springiness	0.75 ± 0.14 ^a^_W_	0.40 ± 0.03 ^c^_WX_	0.52 ± 0.08 ^b^_W_	0.54 ± 0.07 ^b^_XY_	0.53 ± 0.10 ^b^_YZ_
Chewiness	48.42 ± 9.95 ^a^_WX_	19.55 ± 6.65 ^bc^_XY_	29.16 ± 8.19 ^b^_W_	29.13 ± 7.93 ^b^_XY_	18.36 ± 4.25 ^c^_Y_
Cooking time = 30 min					
Hardness	121.07 ± 11.69 ^a^_Y_	79.39 ± 11.91 ^b^_Z_	127.89 ± 10.86 ^a^_X_	121.08 ± 18.34 ^a^_X_	72.23 ± 15.13 ^b^_Z_
Cohesiveness	0.43 ± 0.05 ^ab^_W_	0.41 ± 0.04 ^abc^_X_	0.36 ± 0.02 ^bc^_W_	0.35 ± 0.04 ^c^_Y_	0.44 ± 0.05 ^a^_X_
Springiness	0.72 ± 0.13 ^a^_W_	0.33 ± 0.03 ^c^_X_	0.52 ± 0.09 ^b^_W_	0.49 ± 0.07 ^b^_Y_	0.47 ± 0.09 ^b^_Z_
Chewiness	32.44 ± 6.95 ^a^_X_	12.40 ± 2.60 ^c^_Y_	24.68 ± 6.36 ^ab^_W_	16.26 ± 6.46 ^bc^_Y_	15.34 ± 3.31 ^c^_Y_

Data are presented as mean ± standard deviation (*n* = 15). Means with different lowercase letters in superscripts indicate a significant difference (*p* < 0.05) in TPA texture properties between vegan pasta made with varying PPI levels, when cooked within the same duration. Means with different uppercase letters in subscripts indicate a significant difference (*p* < 0.05) in TPA texture properties between vegan pasta made with the same PPI level but cooked for different durations.

**Table 4:** Results of independent t-test for the perceived firmness, brittleness, and stickiness of PPI0 and PPI25 by fast (chewing time < 13 s) and slow (chewing time > 13 s) chewers.

Chewer Group	PPI Level	Perceived Firmness			Perceived Brittleness			Perceived Stickiness		
		Mean	t	*p*-Value	Mean	t	*p*-Value	Mean	t	*p*-Value
Fast chewers (*n* = 59)	PPI0	51.7	4.1	<0.001	32.2	−1.2	0.225	56.5	4.9	<0.001
	PPI25	35.3			38.2			35.9		
Slow chewers (*n* = 57)	PPI0	48.4	2.8	0.007	33.4	−3.2	0.002	56.6	5.6	<0.001
	PPI25	37.2			48.4			32.7		

## Data Availability

The original contributions presented in the study are included in the article/Supplementary Material, further inquiries can be directed to the corresponding author.

## Supplementary Materials

The following supporting information can be downloaded at: https://www.mdpi.com/article/10.3390/foods13233798/s1, Figure S1: Pasta samples (PPI35) sheeted at 1.3, 2.1, and 2.9 mm using Fimar machine (A); Pasta samples (PPI35, thickness = 2.1 mm) produced at water to dry ingredients ratios (*w*/*w*) of 4:10, 5:10, and 6:10 (B); Pasta samples (thickness = 2.1 mm, water to powder ratio = 6:10) produced at WF to PPI ratios (*w*/*w*) of 100:0, 95:5, 85:15, 75:25, 65:35, and 55:45 (C,D). Figure S2: Optimum cooking time analysis of cooked pasta samples: PPI0, PPI25, and PPI35 after being squeezed by two glass slices according to AACC Method 66–50. Figure S3: Pasta samples (PPI0–35) being cooked for 20, 25, and 30 min after double compression (TPA) analysis. Figure S4: Three-dimensional mesh plot of the TPA: hardness (A), cohesiveness (B), springiness (C), and chewiness (D) of vegan pasta substituted with 0–35% PPI after 5, 10, 15, 20, 25, and 30 min of cooking. Figure S5: Serving trays presented to the participants (A) and in vivo study booth setup (B). The serving tray consists of two sample containers (20 mL) containing samples (d = 20 mm, green arrow), spitting container (blue arrow), digital timer (red arrow), plastic spoon (yellow arrow), and tissue paper (purple arrow). Figure S6: Average moisture content of pasta samples before and after cooking for 5, 10, 15, and 20 min. Table S1: Color properties of vegan pasta at different PPI substitution levels when cooked between 5 and 20 min.
