# Supplementary material for: Texture Evaluation and In Vivo Oral Tactile Perceptions of Cooked Wheat Pasta Sheets Partially Substituted with Pea Protein
Source: Foods. 2024 Nov 26;13(23):3798. doi: 10.3390/foods13233798 (PMC11640413; doi:10.3390/foods13233798)
Supplement: Supplementary file 1 [file foods-13-03798-s001.zip › foods-3333878-supplementary.pdf]

foods

Supplementary Materials

Texture Evaluation and In Vivo Oral Tactile Perceptions of Cooked Wheat Pasta Sheets Partially Substituted with Pea Pro-tein

Chengyi Yang [1,2,3], Sze Ying Leong [1,2], Jessie King [1,2], Esther H.-J. Kim [2,3], Marco P. Morgenstern [2,3], Mei Peng [1,2], Dominic Agyei [1], Kevin Sutton [2,3] and Indrawati Oey [1,2,*]

[1] Department of Food Science, University of Otago, Dunedin 9054, New Zealand; yange860@student.otago.ac.nz (C.Y.); sze.leong@otago.ac.nz (S.Y.L.); jessie.king@otago.ac.nz (J.K.); mei.peng@otago.ac.nz (M.P.); dominic.agyei@otago.ac.nz (D.A.)
[2] Riddet Institute, Palmerston North 4442, New Zealand; esther.kim@plantandfood.co.nz (E.H.-J.K.); marco.morgenstern@plantandfood.co.nz (M.P.M.); kevin.sutton@plantandfood.co.nz (K.S.)
[3] The New Zealand Institute for Plant & Food Research Limited, Lincoln 7608, New Zealand
* Correspondence: indrawati.oey@otago.ac.nz

Figure S1: Pasta samples (PPI35) sheeted at 1.3, 2.1, and 2.9 mm using Fimar machine (A); Pasta samples (PPI35, thickness=2.1 mm) produced at water to dry ingredients ratios (w/w) of 4:10, 5:10, and 6:10 (B); Pasta samples (thickness=2.1 mm, water to powder ratio=6:10) produced at WF to PPI ratios (w/w) of 100:0, 95:5, 85:15, 75:25, 65:35, and 55:45 (C,D).

Figure S2: Optimum cooking time analysis of cooked pasta samples: PPI0, PPI25, and PPI35 after being squeezed by two glass slices according to AACC Method 66-50.

Figure S3: Pasta samples (PPI0-35) being cooked for 20, 25, and 30 min after double compression (TPA) analysis.

Figure S4: Three-dimensional mesh plot of the TPA: hardness (A), cohesiveness (B), springiness (C), gumminess (D), and chewiness (E) of vegan pasta substituted with 0-35% PPI after 5, 10, 15, 20, 25, and 30 min of cooking.

Figure S5: Serving trays presented to the participants (A) and in vivo study booth setup (B). The serving tray consists of two sample containers (20 mL) containing samples (d=20 mm, green arrow), spitting container (blue arrow), digital timer (red arrow), plastic spoon (yellow arrow), and tissue paper (purple arrow).

Figure S6: Average moisture content of pasta samples before and after cooking for 5, 10, 15, 20 min.

Table S1: Color properties of vegan pasta at different PPI substitution levels when cooked between 5 and 20 min.

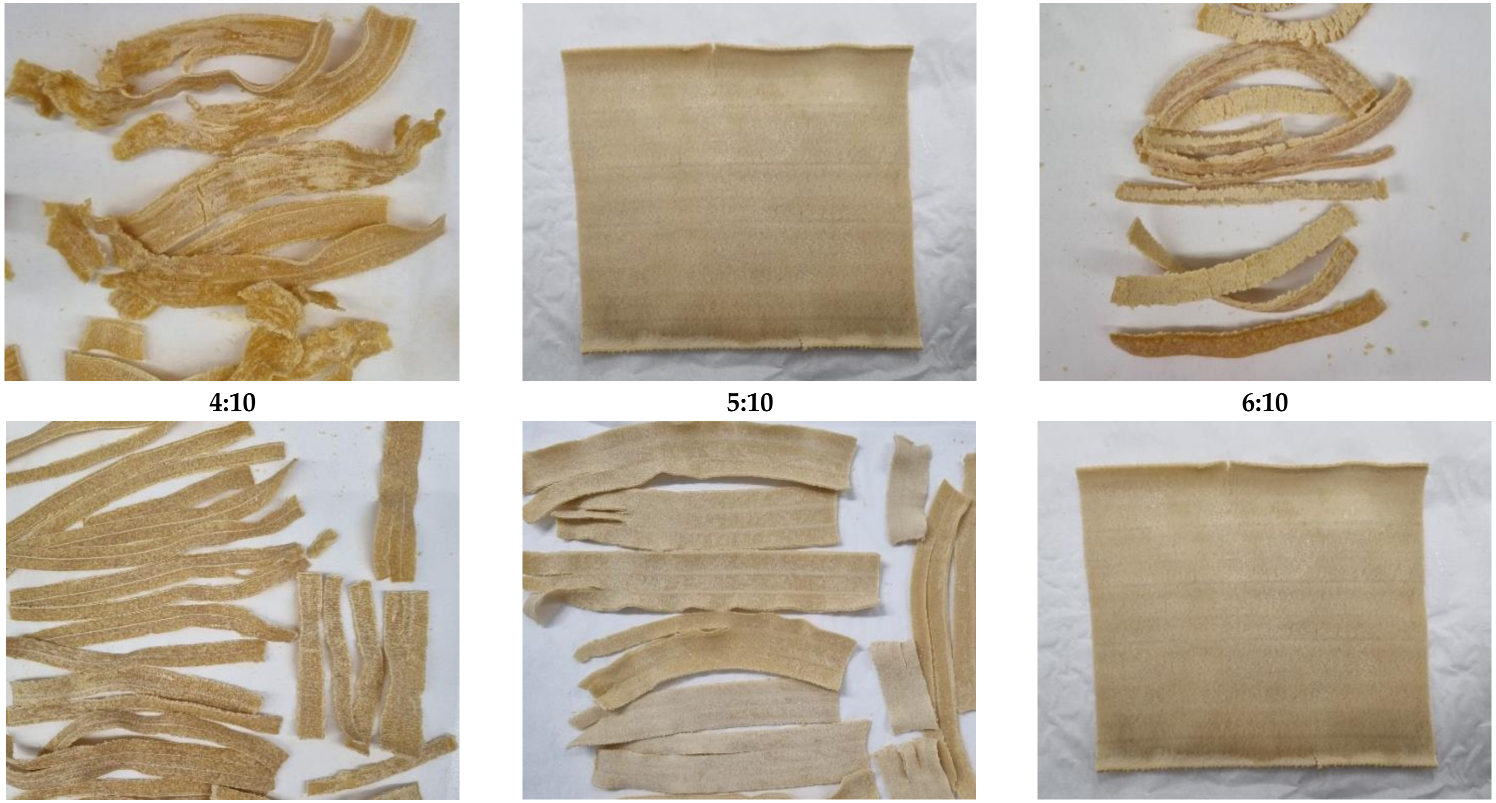
Sheet thickness
1.3 mm
2.1 mm
2.9 mm
A
Water to powder ratio
4:10
5:10
6:10
B

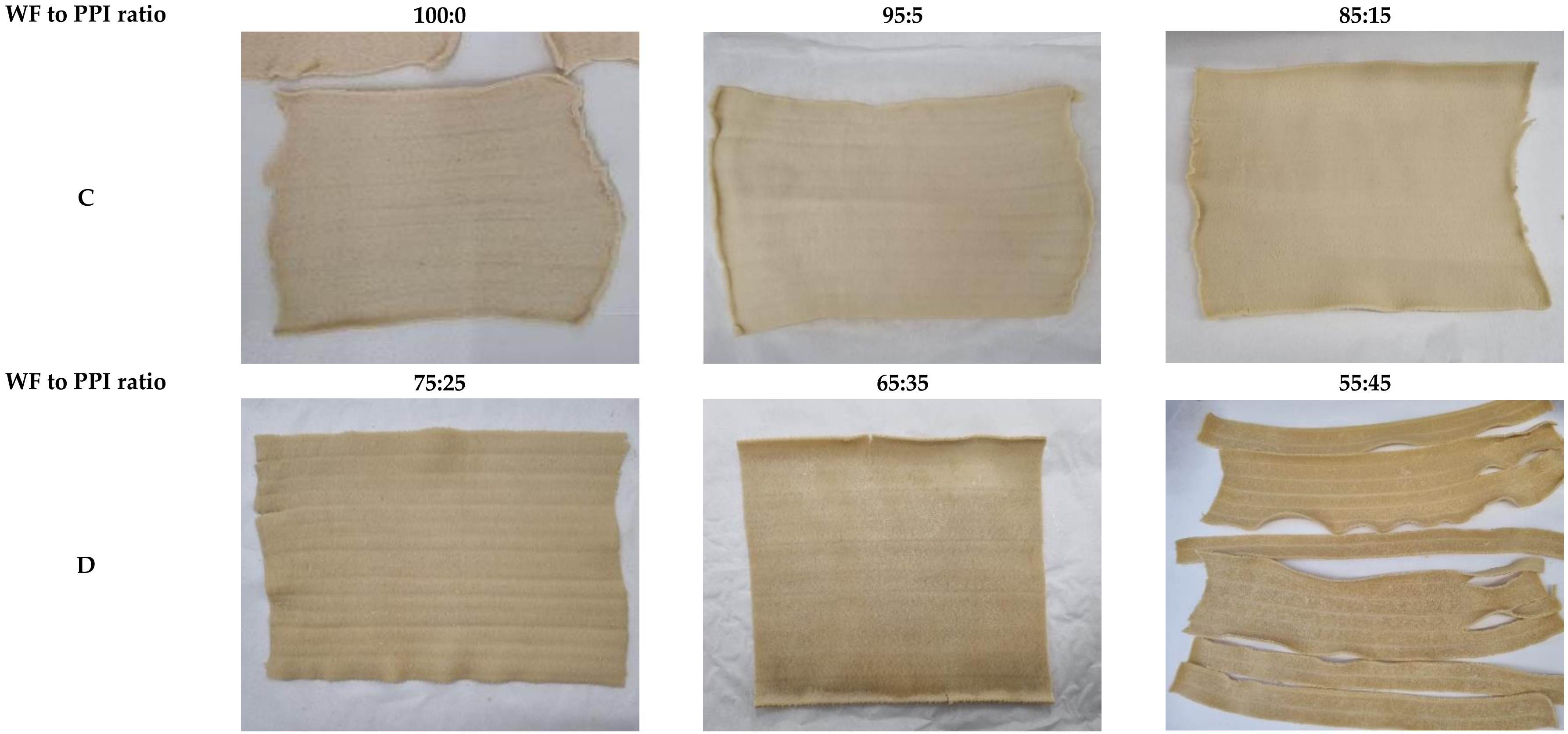

Figure S1. Pasta samples (PPI35) sheeted at 1.3, 2.1, and 2.9 mm using Fimar machine (**A**); Pasta samples (PPI35, thickness=2.1 mm) produced at water to dry ingredients ratios (w/w) of 4:10, 5:10, and 6:10 (**B**); Pasta samples (thickness=2.1 mm, water to powder ratio=6:10) produced at WF to PPI ratios (w/w) of 100:0, 95:5, 85:15, 75:25, 65:35, and 55:45 (**C,D**).

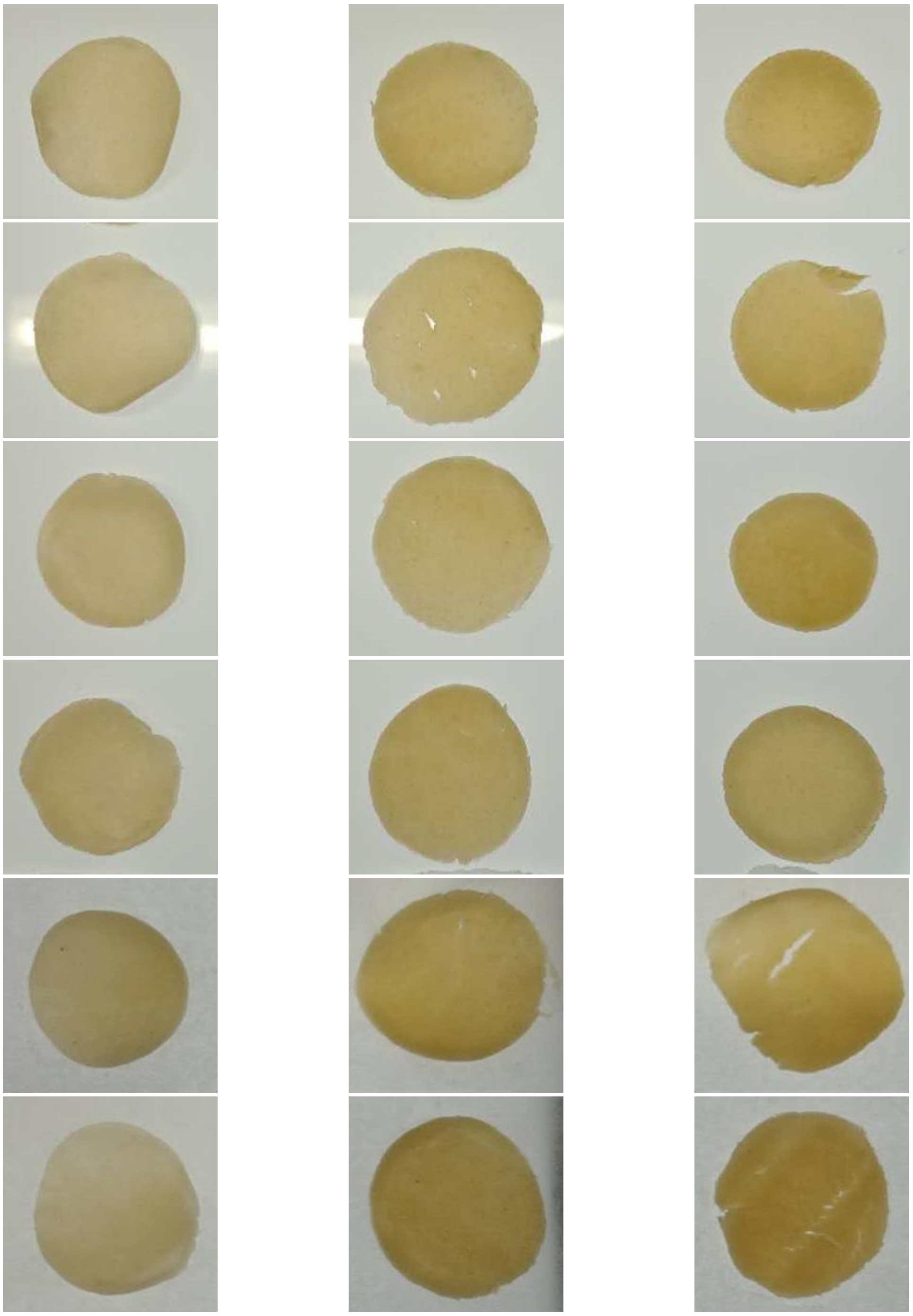

Figure S2. Optimum cooking time analysis of cooked pasta samples: PPI0, PPI25, and PPI35 after being squeezed by two glass slices according to AACC Method 66-50.

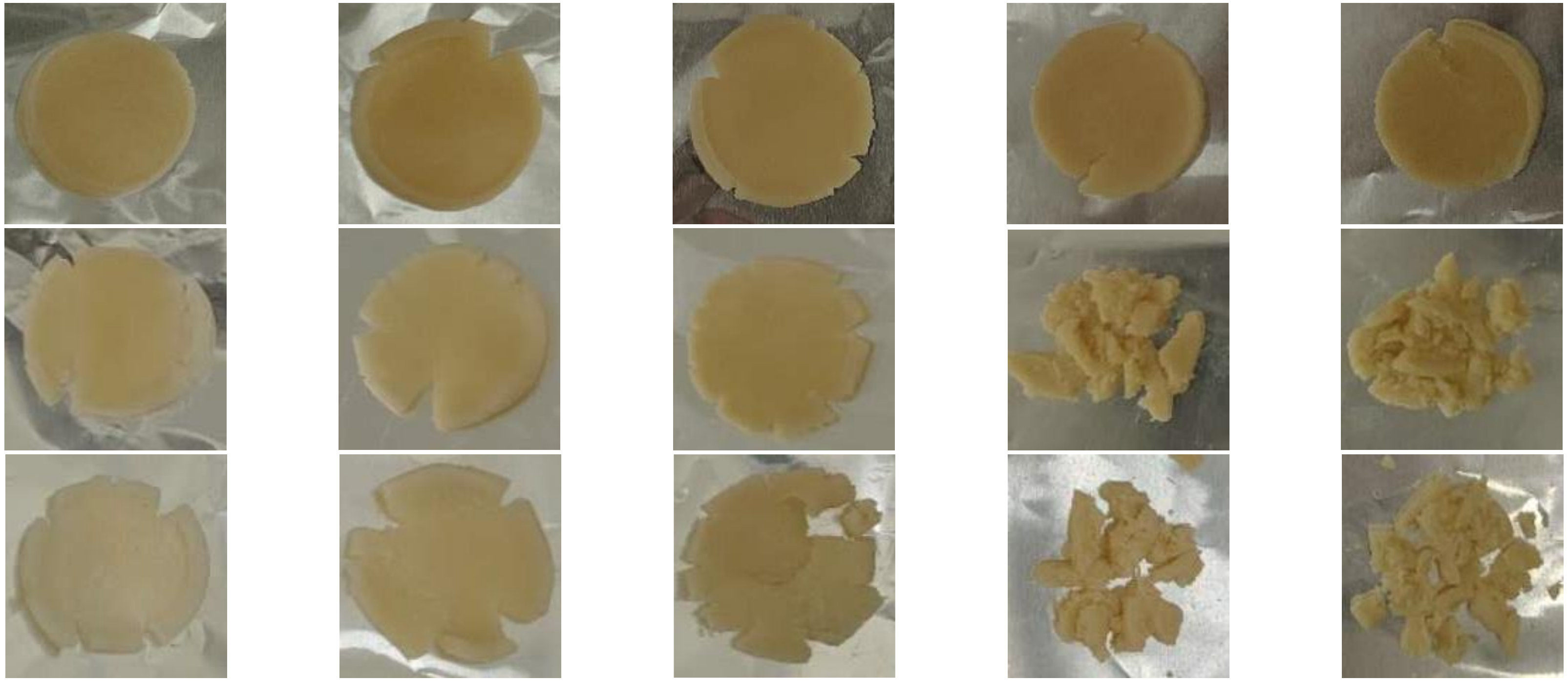

Figure S3. Pasta samples (PPI0-35) being cooked for 20, 25, and 30 min after double compression (TPA) analysis.

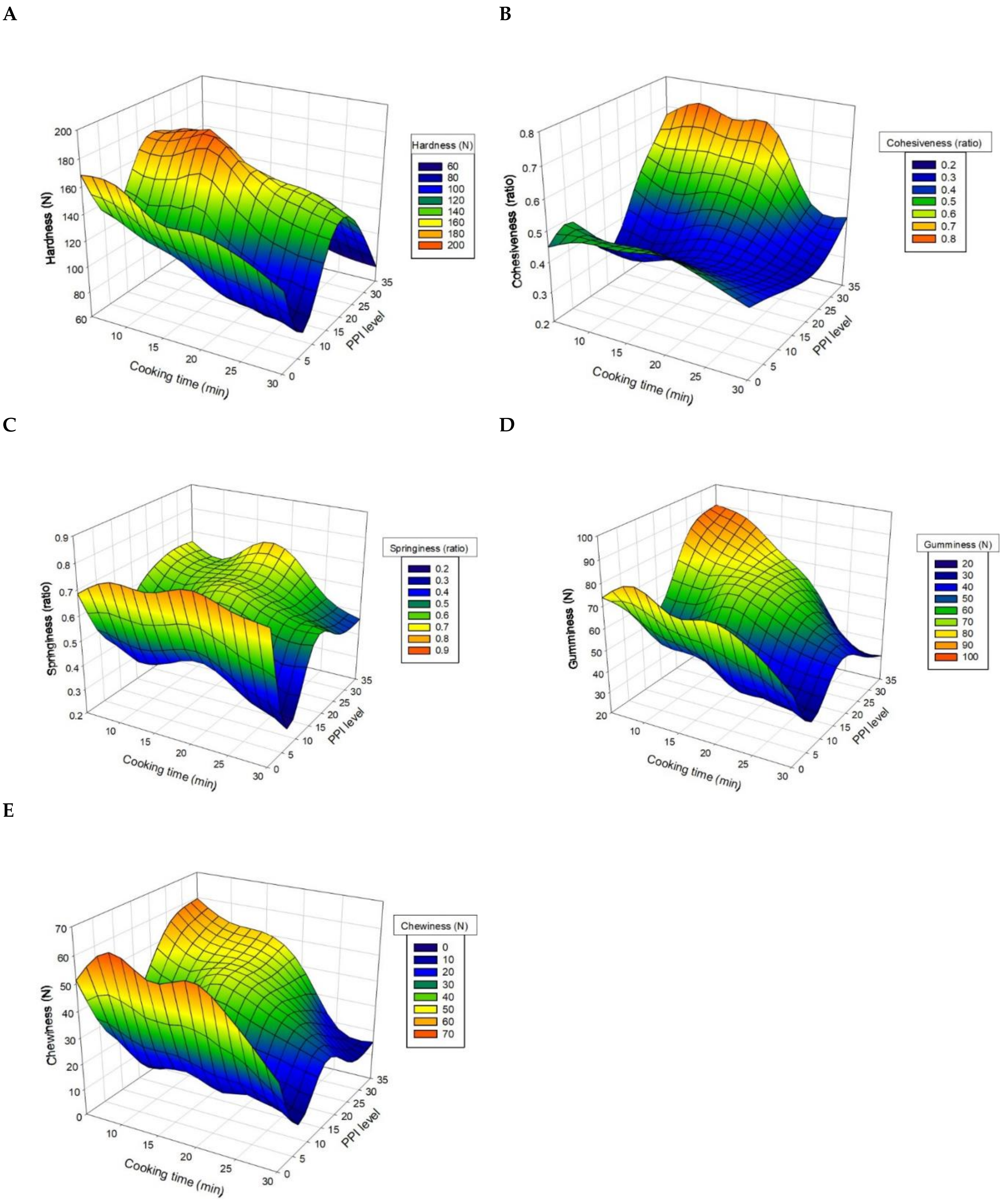

Figure S4. Three-dimensional mesh plot of the TPA: hardness (**A**), cohesiveness (**B**), springiness (**C**), gumminess (**D**), and chewiness (**E**) of vegan pasta substituted with 0-35% PPI after 5, 10, 15, 20, 25, and 30 min of cooking.

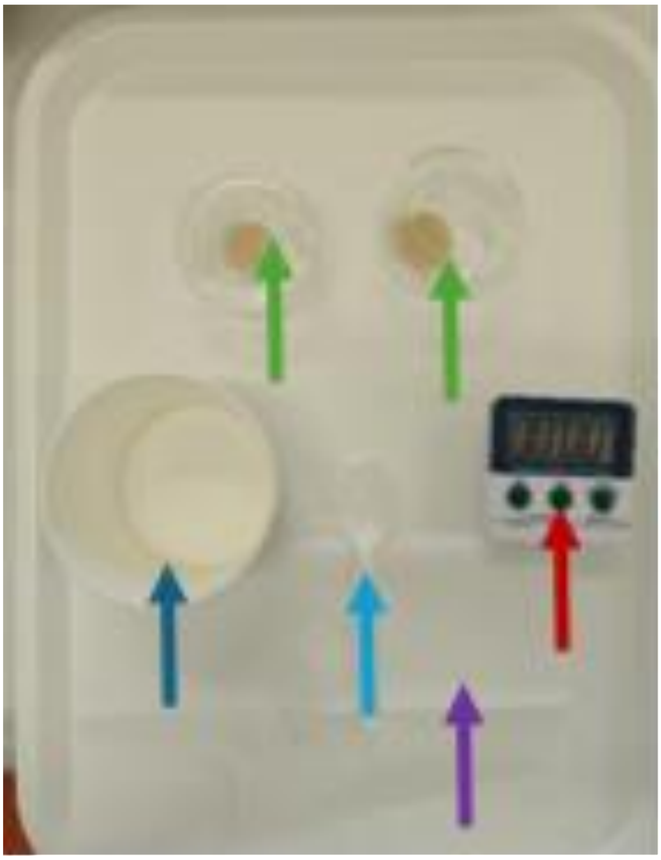
A

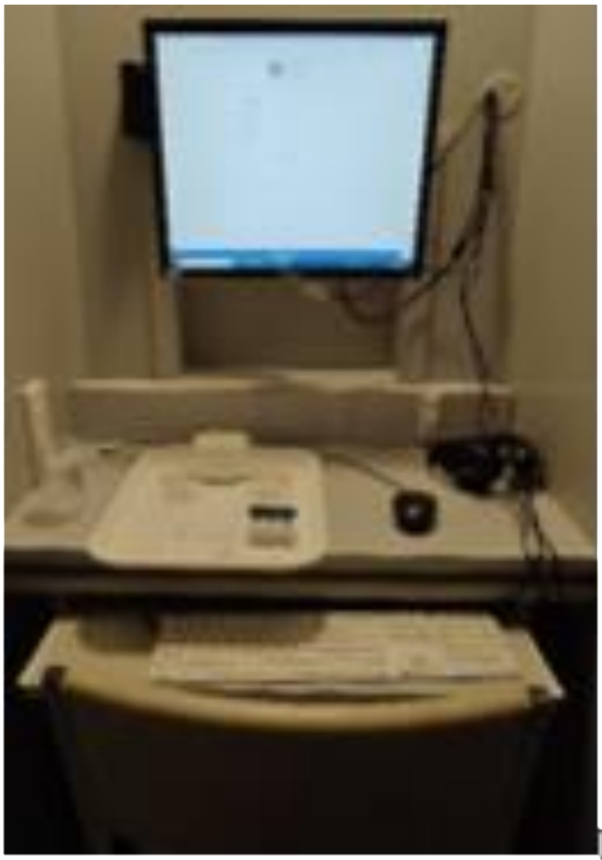
B

Figure S5. Serving trays presented to the participants (**A**) and in vivo study booth setup (**B**). The serving tray consists of two sample containers (20 mL) containing samples (d=20 mm, green arrow), spitting container (blue arrow), digital timer (red arrow), plastic spoon (yellow arrow), and tissue paper (purple arrow).

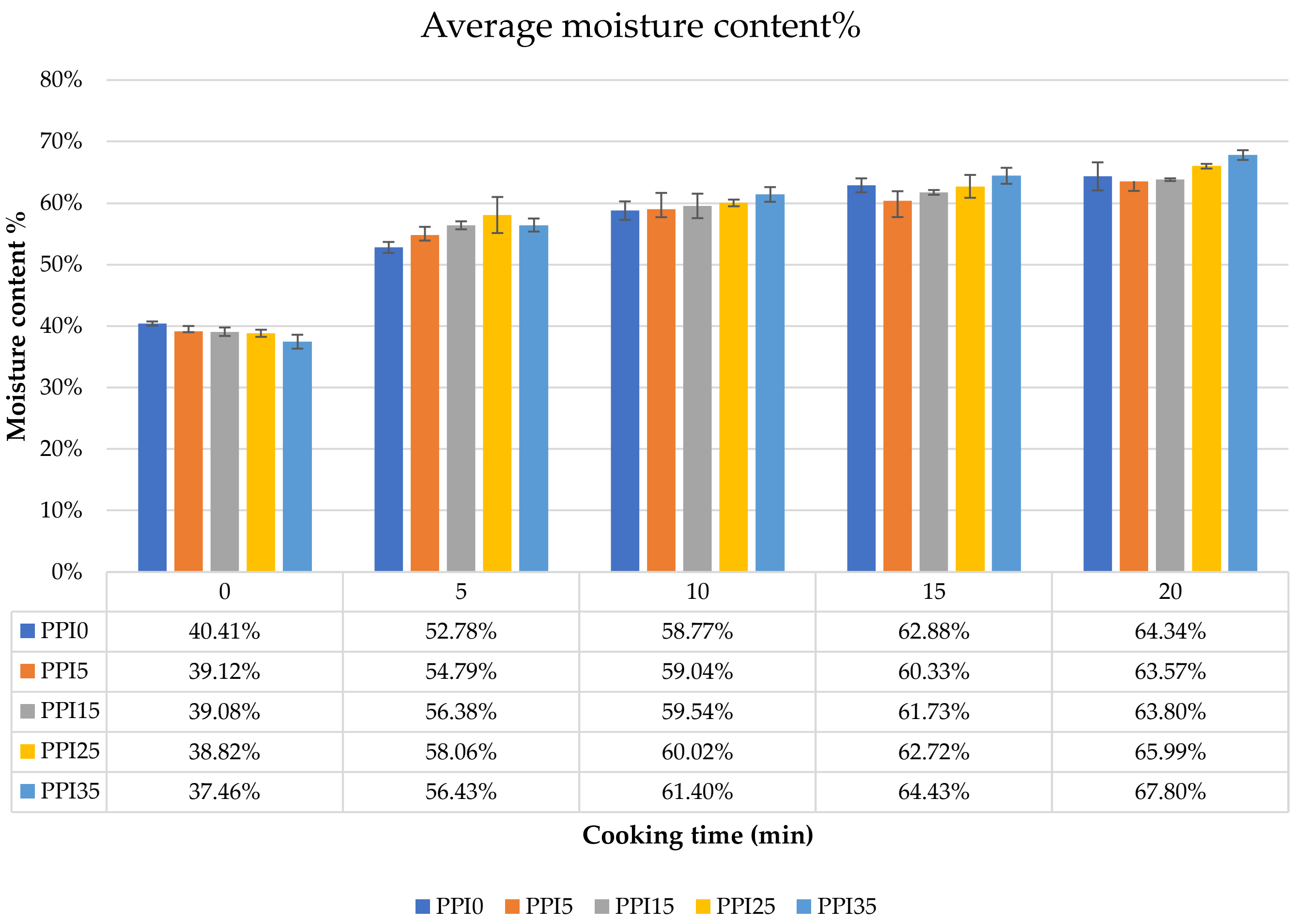

	0	5	10	15	20
PPI0	40.41%	52.78%	58.77%	62.88%	64.34%
PPI5	39.12%	54.79%	59.04%	60.33%	63.57%
PPI15	39.08%	56.38%	59.54%	61.73%	63.80%
PPI25	38.82%	58.06%	60.02%	62.72%	65.99%
PPI35	37.46%	56.43%	61.40%	64.43%	67.80%

Figure S6. Average moisture content of pasta samples before and after cooking for 5, 10, 15, 20 min.

Table S1. Color properties of vegan pasta at different PPI substitution levels when cooked between 5 and 20 min.

Color properties (n=3)	PPI substitution levels				
	0% (control)	5%	15%	25%	35%
			Cooking time = 5 min		
L*	$48.43 \pm 0.36^{a}_{Y}$	$48.37 \pm 0.63^{a}_{Y}$	$48.06 \pm 0.46^{a}_{XY}$	$46.00 \pm 0.46^{b}_{Z}$	$46.16 \pm 0.44^{b}_{Y}$
a*	$2.04 \pm 0.11^{d}_{XY}$	$2.49 \pm 0.18^{c}_{X}$	$2.46 \pm 0.15^{c}_{Y}$	$2.79 \pm 0.14^{b}_{X}$	$3.36 \pm 0.18^{a}_{X}$
b*	$13.41 \pm 0.34^{d}_{X}$	$15.34 \pm 0.61^{c}_{X}$	$15.29 \pm 0.40^{c}_{Z}$	$15.91 \pm 0.52^{b}_{X}$	$17.16 \pm 0.27^{a}_{X}$
Chroma	$13.56 \pm 0.34^{d}_{X}$	$15.54 \pm 0.63^{c}_{X}$	$15.48 \pm 0.42^{c}_{Z}$	$16.15 \pm 0.53^{b}_{X}$	$17.49 \pm 0.28^{a}_{X}$
Hue	$81.35 \pm 0.33^{a}_{Y}$	$80.78 \pm 0.39^{b}_{X}$	$80.87 \pm 0.35^{b}_{Y}$	$80.14 \pm 0.30^{c}_{Z}$	$78.93 \pm 0.50^{d}_{Z}$
Browning index	$34.87 \pm 1.19^{d}_{X}$	$41.17 \pm 2.38^{c}_{X}$	$41.28 \pm 1.79^{c}_{Z}$	$46.00 \pm 2.23^{b}_{Y}$	$50.85 \pm 1.41^{a}_{Y}$
			Cooking time = 10 min		
L*	$48.44 \pm 0.33^{a}_{Y}$	$48.59 \pm 0.35^{a}_{XY}$	$47.76 \pm 0.20^{b}_{Y}$	$46.58 \pm 0.41^{c}_{Y}$	$46.78 \pm 0.60^{c}_{Y}$
a*	$2.18 \pm 0.16^{c}_{X}$	$2.49 \pm 0.20^{b}_{X}$	$2.57 \pm 0.18^{b}_{XY}$	$2.61 \pm 0.16^{b}_{X}$	$3.27 \pm 0.16^{a}_{XY}$
b*	$13.84 \pm 0.41^{d}_{X}$	$15.68 \pm 0.83^{cd}_{X}$	$16.25 \pm 0.76^{b}_{Y}$	$15.63 \pm 0.70^{c}_{X}$	$17.12 \pm 0.33^{a}_{X}$
Chroma	$14.01 \pm 0.42^{d}_{X}$	$15.88 \pm 0.85^{cd}_{X}$	$16.45 \pm 0.78^{b}_{Y}$	$15.85 \pm 0.72^{c}_{X}$	$17.43 \pm 0.35^{a}_{X}$
Hue	$81.03 \pm 0.50^{a}_{XY}$	$80.98 \pm 0.34^{a}_{X}$	$81.01 \pm 0.28^{a}_{XY}$	$80.53 \pm 0.19^{b}_{Y}$	$79.19 \pm 0.40^{c}_{Z}$
Browning index	$36.29 \pm 1.33^{d}_{X}$	$41.97 \pm 2.90^{c}_{X}$	$44.69 \pm 2.65^{c}_{Y}$	$44.20 \pm 2.85^{b}_{XY}$	$49.75 \pm 1.92^{a}_{XY}$
			Cooking time = 15 min		
L*	$48.73 \pm 0.54^{a}_{XY}$	$49.09 \pm 0.37^{a}_{Y}$	$47.76 \pm 0.33^{a}_{Y}$	$47.56 \pm 0.31^{b}_{X}$	$47.55 \pm 0.67^{b}_{X}$
a*	$2.06 \pm 0.10^{d}_{XY}$	$2.45 \pm 0.15^{c}_{X}$	$2.70 \pm 0.08^{b}_{X}$	$2.44 \pm 0.13^{c}_{Y}$	$3.13 \pm 0.20^{a}_{YZ}$
b*	$13.74 \pm 1.18^{c}_{X}$	$15.62 \pm 1.21^{b}_{X}$	$17.02 \pm 1.40^{a}_{X}$	$15.74 \pm 1.23^{a}_{X}$	$17.05 \pm 1.45^{a}_{X}$
Chroma	$13.89 \pm 1.21^{d}_{X}$	$15.81 \pm 1.25^{c}_{X}$	$17.24 \pm 1.43^{b}_{X}$	$15.93 \pm 1.26^{c}_{X}$	$17.34 \pm 1.50^{a}_{X}$
Hue	$81.47 \pm 0.93^{a}_{XY}$	$81.10 \pm 0.91^{b}_{X}$	$80.99 \pm 0.91^{b}_{XY}$	$81.20 \pm 0.93^{b}_{X}$	$79.60 \pm 1.07^{c}_{XY}$
Browning index	$35.58 \pm 5.11^{c}_{X}$	$41.19 \pm 5.07^{b}_{X}$	$47.27 \pm 5.68^{a}_{X}$	$43.14 \pm 5.27^{b}_{XY}$	$38.37 \pm 6.05^{a}_{XY}$
			Cooking time = 20 min		
L*	$49.29 \pm 0.56^{a}_{X}$	$48.69 \pm 0.48^{ab}_{XY}$	$48.34 \pm 0.60^{bc}_{X}$	$47.88 \pm 0.37^{cd}_{X}$	$47.69 \pm 0.25^{d}_{X}$
a*	$1.98 \pm 0.14^{d}_{Y}$	$2.48 \pm 0.14^{cd}_{X}$	$2.59 \pm 0.14^{b}_{XY}$	$2.37 \pm 0.15^{c}_{Y}$	$3.01 \pm 0.16^{a}_{Z}$
b*	$13.74 \pm 0.47^{d}_{X}$	$15.94 \pm 0.67^{c}_{X}$	$16.88 \pm 0.64^{b}_{XY}$	$15.72 \pm 0.89^{c}_{X}$	$17.00 \pm 0.69^{a}_{X}$
Chroma	$13.88 \pm 0.48^{c}_{X}$	$16.13 \pm 0.68^{b}_{X}$	$17.08 \pm 0.66^{a}_{XY}$	$15.90 \pm 0.90^{b}_{X}$	$17.26 \pm 0.70^{a}_{X}$
Hue	$81.80 \pm 0.46^{a}_{X}$	$81.18 \pm 0.24^{b}_{X}$	$81.30 \pm 0.22^{b}_{X}$	$81.42 \pm 0.19^{b}_{X}$	$79.95 \pm 0.31^{c}_{X}$
Browning index	$34.98 \pm 1.60^{c}_{X}$	$42.60 \pm 2.37^{b}_{X}$	$45.97 \pm 1.95^{a}_{XY}$	$42.66 \pm 3.08^{b}_{X}$	$47.79 \pm 2.35^{a}_{X}$

Data are presented as mean ± standard deviation (n=3). Means with different lowercase letters in superscripts indicate a significant difference ($p<0.05$) in color properties between vegan pasta made with varying PPI levels, when cooked within the same duration. Means with different uppercase letters in subscripts indicate a significant difference ($p<0.05$) in color properties between vegan pasta made with same PPI level, but cooked for different durations.
